# Synthesis and *In Vitro* Antibacterial Activity of 7-(3-Amino-6,7-dihydro-2-methyl-2*H*-pyrazolo[4,3-c] Pyridin-5(4*H*)-yl)fluoroquinolone Derivatives

**DOI:** 10.3390/molecules16032626

**Published:** 2011-03-22

**Authors:** Xin Guo, Ming Liang Liu, Hui Yuan Guo, Yu Cheng Wang, Ju Xian Wang

**Affiliations:** 1 Institute of Medicinal Biotechnology, Chinese Academy of Medical Sciences and Peking Union Medical College, Beijing 100050, China; 2 College of Pharmaceutical Sciences, Southwest University, Chongqing 400715, China; E-Mail:guoxincq@hotmail.com

**Keywords:** fluoroquinolone, antibacterial activity, synthesis

## Abstract

A series of novel 7-(3-amino-6,7-dihydro-2-methyl-2*H*-pyrazolo[4,3-c]pyridin- 5(4*H*)-yl)fluoroquinolone derivatives were designed, synthesized and characterized by ^1^H-NMR, MS and HRMS. These fluoroquinolones were evaluated for their *in vitro* antibacterial activity against representative Gram-positive and Gram-negative strains. Results reveal that most of the target compounds exhibit good growth inhibitory potency against methicillin-resistant *Staphylococcus epidermidis* (MRSE) (MIC: 0.25–4 μg/mL) and *Streptococcus pneumoniae* (MIC: 0.25–1 μg/mL). In addition, compound **8f **is 8–128 fold more potent than the reference drugs gemifloxacin (GM), moxifloxacin (MX), ciprofloxacin (CP) and levofloxacin (LV) against methicillin-resistant *Staphylococcus aureus* 10-05 and *Streptococcus hemolyticus* 1002 and 2–64 fold more active against methicillin-sensitive *Staphylococcus aureus* 10-03 and 10-04.

## 1. Introduction

Since the discovery of nalidixic acid by Lesher *et al.* in 1962 [1], the quinolones have evolved into an important class of antibacterial agents used mainly for the treatment of respiratory tract infections (RTI), urinary tract infections (UTI), sexually transmitted diseases (STD), gastrointestinal and abdominal infections, skin and soft tissue infections, and infections of the bone and joints, among many other uses [2]. These compounds act by binding to the quinolone-resistance-determining region (QRDR) in the catalytic domain of the topoisomerase II (DNA gyrase) or IV complex with DNA. Cell death is induced by trapping the topoisomerase protein-DNA complex thus disrupting normal DNA replication, inducing oxidative damage, and triggering cell-death mechanisms. DNA gyrase appears to be the primary target for quinolones in Gram-negative bacteria such as *Escherichia coli*, while topoisomerase IV is the primary target in Gram-positive bacteria such as *Staphylococcus aureus* [3,4].

Despite the large number of fluoroquinolones approved for the treatment of bacterial infections, there have been unabated efforts for the discovery of new quinolones with specific improved properties and most importantly, to overcome the growing problem of bacterial resistance. Furthermore, some of the side effects of quinolone antibacterials are unacceptable, for example, grepafloxacin was withdrawn from market due to increased cases of heart problems in clinical findings [5]. Similarly, Trovafloxacin was removed from the market due to its liver toxicity [5].

In a search for potent fluoroquinolone derivatives, it has been found that although some were based on modifications on other positions, the most successful compounds developed were based on modifications at C-7, and it has been found that the spectrum and level of antibacterial activity is highly affected by the nature of the C-7 substituent group [6]. In general, 5- and 6-membered nitrogen heterocycles including piperazinyl, pyrrolidinyl and piperidinyl type side chains have been proven to be the optimal substituents [7].

**Figure 1:**
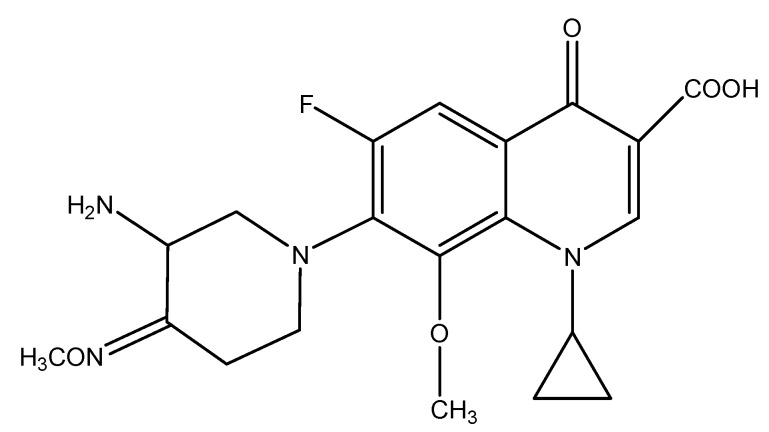
New 8-methoxylfluoroquinolone IMB.

As part of an ongoing program to find potent and broad-spectrum antibacterial agents that display strong Gram-positive activities, we also have focused on introducing new functional groups to the piperidine ring [8,9,10,11,12]. Interestingly, IMB (Figure 1), a new 8-methoxylfluoroquinolone incorporating a 3-amino-4-methoxyiminopiperidine at C-7 position, shows excellent *in vitro* and *in vivo* antibacterial activities [8]. 

In this paper a series of novel fluoroquinolone compounds containing both the piperidine ring and the 3-aminopyrazole ring at the 7-position were designed and synthesized. The 3-aminopyrazole function group at the 3-positions of some fourth generation cephalosporins, such as cefoselis, was fused with a piperidine to furnish the 4,5,6,7-tetrahydro-2-methyl-2*H*-pyrazolo[4,3-c] pyridin-3-amine scaffold which can be readily obtained from the corresponding 4-oxopiperidine-3-carbonitrile in a single operation. Our primary objective was to optimize the potency of these compounds against Gram-positive and Gram-negative organisms.

## 2. Results and Discussion

### 2.1. Synthesis of 7-(3-amino-6,7-dihydro-2-methyl-2H-pyrazolo[4,3-c]pyridin-5(4H)-yl)fluoro- quinolone derivatives

**Scheme 1:**
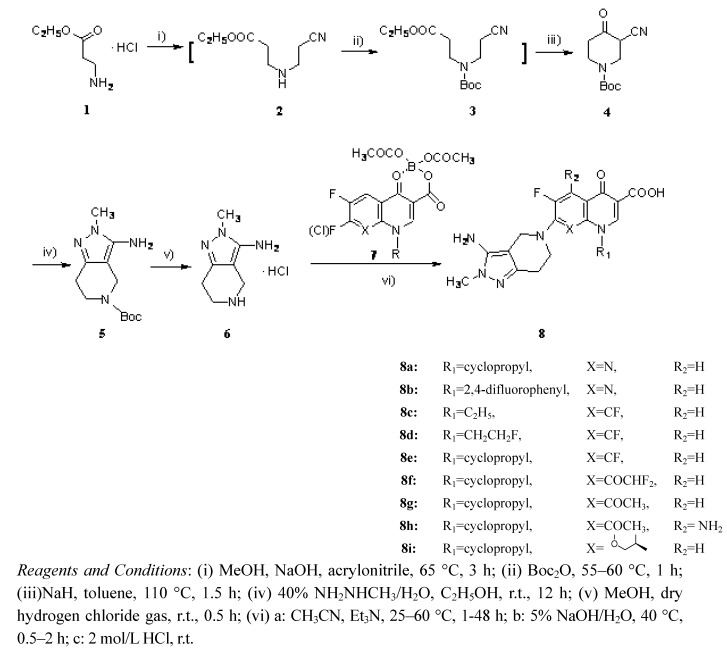
Synthesis of novel fluoroquinolones **8a-i**.

The synthesis of novel fluoroquinolone derivatives **8a-i** is outlined in Scheme 1. Addition reaction of ethyl 3-aminopropanoate hydrochloride (**1**) with acrylonitrile in the presence of sodium hydroxide gave the secondary amine **2**, which was subsequently treated with di-*tert*-butoxycarbonyl dicarbonate (Boc_2_O) to produce Boc-protected cyano ester **3**. 

Compound **3** was cyclized to the cyanoketone **4 ** by sodium hydride in refluxing toluene (overall yield of 83% for the three steps). Compound **4** was treated with methylhydrazine in ethanol to give the Boc-protected bicyclic amines **5**, which upon deprotection gave the key intermediates **6** after treatment with hydrogen chloride gas [13,14]. Finally, the target compounds **8a-i** were obtained by coupling the intermediates **6 **with various boric acid chelates **7 ** containing quinolone and naphthyridone cores**, **and then hydrolysis of the chelating groups according to well-established literature procedures [11,12,15].

### 2.2. Antibacterial activity of 7-(3-amino-6,7-dihydro-2-methyl-2H-pyrazolo[4,3-c]pyridin-5(4H)-yl)- fluoroquinolone derivatives

The *in vitro* antibacterial activity of the novel fluoroquinolones **8a-i **against representative Gram-negative and Gram-positive strains was evaluated using standard techniques [16]. Minimum inhibitory concentration (MIC) of the synthesized compounds (the concentration of the compound required to cause complete inhibition of bacterial growth), along with those of the standard drugs gemifloxacin (GM), moxifloxacin (MX), ciprofloxacin (CP) and levofloxacin (LV) are given in Table 1.

**Table 1:** *In-vitro* antibacterial activity of compounds **8a-i**.

Strains	Compd. MIC (μg/mL)^ a)^												
	8a	8b	8c	8d	8e	8f	8g	8h	8i	GM	MX	LV	CP
*S.a.*1	0.5	0.25	0.25	0.5	0.25	0.25	1	2	0.25	0.008	0.03	0.125	0.25
*S.a.*2	128	>128	>128	>128	128	16	64	128	128	32	8	32	64
*S.a.*3	64	4	0.5	0.25	128	0.5	1	64	32	0.5	1	8	0.25
*S.a.*4	128	128	128	128	>128	16	32	64	16	2	0.125	0.125	0.25
*S.a.*5	16	2	4	128	128	0.5	2	128	128	4	8	32	64
*S.a.*6	64	0.25	16	128	8	0.5	32	64	16	16	0.125	0.125	0.25
*S.a.*7	4	128	16	128	32	4	32	64	32	8	4	4	32
*S.a.*8	8	64	32	128	8	0.5	8	16	8	1	4	8	1
*S.a.*9	16	128	16	2	4	0.25	4	8	2	2	8	8	16
*S.a.*10	8	64	32	128	8	4	8	64	2	0.5	4	8	1
*S.e.*1	1	4	32	16	1	0.25	1	32	0.25	0.03	0.06	0.06	0.25
*S.e.*2	1	0.25	1	0.25	0.25	0.5	0.25	0.25	2	0.008	0.06	0.125	2
*S.e.*3	0.5	2	4	0.5	0.5	0.5	0.5	0.5	0.5	0.06	0.125	0.5	4
*S.e.*4	0.25	0.5	2	1	2	2	2	1	2	0.125	0.25	1	2
*S.e.*5	2	1	2	4	2	4	4	2	1	8	4	8	16
*S.e.*6	64	16	1	0.25	128	16	64	32	128	1	4	4	0.03
*S.e.*7	128	>128	8	4	64	4	128	128	128	0.25	8	8	16
*S.e.*8	128	16	2	0.5	128	4	32	16	128	0.5	4	4	2
*S.e9*	64	128	1	0.25	128	16	64	64	128	0.25	0.5	2	1
*S.e.*10	32	64	2	2	128	2	16	16	128	0.125	0.25	4	8
*S.p.*1	0.25	0.25	0.25	0.25	0.25	0.25	0.25	0.25	0.25	0.008	0.008	0.008	0.06
*S.p.*2	0.5	0.25	0.25	0.25	0.25	0.25	1	0.25	0.25	0.008	0.03	0.008	0.008
*S.p.*3	1	0.5	32	0.5	32	0.5	0.5	0.5	0.5	0.03	0.03	0.015	0.03
*S.h.*1	64	128	32	128	32	64	32	128	16	0.03	0.125	0.125	0.06
*S.h.*2	64	>128	>128	>128	128	0.5	32	128	128	8	4	32	64
*E.c.*1	1	4	1	2	0.25	0.25	2	64	0.25	0.008	0.008	0.008	0.008
*E.c.*2	1	8	1	0.25	0.25	0.5	2	64	0.25	0.06	0.5	0.5	0.25
*E.c.*3	128	128	32	128	128	64	128	128	128	2	16	32	32
*E.c.*4	128	128	128	128	32	64	128	128	128	8	4	2	8
*K.p.*1	>128	>128	>128	>128	64	128	>128	128	>128	32	16	16	32
*K.p.*2	>128	>128	>128	>128	128	128	>128	128	>128	4	4	8	16
*K.p.*3	>128	>128	>128	>128	128	128	>128	128	>128	4	4	4	8
*P.a.*1	>128	>128	128	128	128	128	>128	128	128	4	2	8	64
*P.a.*2	>128	>128	>128	>128	128	128	>128	128	>128	2	8	16	8
*P.a.*3	>128	>128	>128	>128	128	128	>128	128	>128	16	4	32	128

^a)^ The values were reproduced in three experiments; ^b)^Abbreviations: *S.a*.1, *Staphylococcus aureus* ATCC259223; *S.a*.2, methicillin-resistant *Staphylococcus aureus* 10-02; *S.a*.3, methicillin-resistant *Staphylococcus aureus* 10-03; *S.a*.4, methicillin-resistant *Staphylococcus aureus* 10-04; *S.a*.5, methicillin-resistant *Staphylococcus aureus* 10-05; *S.a*.6, methicillin-sensitive *Staphylococcus aureus* 10-01; *S.a*.7, methicillin-sensitive *Staphylococcus aureus* 10-02; *S.a*.8, methicillin-sensitive *Staphylococcus aureus* 10-03; *S.a*.9, methicillin-sensitive *Staphylococcus aureus* 10-04; *S.a*.10, methicillin-sensitive *Staphylococcus aureus* 10-05; *S.e*.1, methicillin-resistant *Staphylococcus epidermidis* 10-1; *S.e*.2, methicillin-resistant *Staphylococcus epidermidis* 10-2; *S.e*.3, methicillin-resistant *Staphylococcus epidermidis* 10-3; *S.e*.4, methicillin-resistant *Staphylococcus epidermidis* 10-4; *S.e*.5, methicillin-resistant *Staphylococcus epidermidis* 10-5;*S.e*.6, methicillin-sensitive *Staphylococcus epidermidis* 10-1; *S.e*.7, methicillin-sensitive *Staphylococcus epidermidis* 10-2; *S.e*.8, methicillin-sensitive *Staphylococcus epidermidis* 10-3; *S.e*.9, methicillin-sensitive *Staphylococcus epidermidis* 10-4; *S.e*.10, methicillin-sensitive *Staphylococcus epidermidis* 10-5; *S.p*.1, *Streptococcus pneumoniae* 1001; *S.p*.2, *Streptococcus pneumoniae* 1003; *S.p*.3, *Streptococcus pneumoniae* 1004; *S.h*.1, *Streptococcus hemolyticus* 1001; *S.h*.2, *Streptococcus hemolyticus* 1002; *E.c*.1, *Escherichia coli* ATCC 25922; *E.c*.2, *Escherichia coli* 10-02; *E.c*.3, *Escherichia coli* 10-03; *E.c*.4, *Escherichia coli* 10-12; *K.p*.1, *Klebsiella* *pneumoniae* 10-1; *K.p*.2, *Klebsiella pneumoniae* 10-2; *K.p*.3, *Klebsiella pneumoniae* 1025; *P.a.*1, *Pseudomonas aeruginosa* ATCC27853; *P.a.*2, *Pseudomonas aeruginosa* 10-2; *P.a.*3, *Pseudomonas aeruginosa* 10-3.

The novel fluoroquinolones **8a-i** display generally rather weak potency against the tested Gram-negative strains, but most of them exhibit good potency in inhibiting the growth of methicillin-resistant *Staphylococcus epidermidis* (MRSE) (MIC: 0.25-4 μg/mL) and *Streptococcus pneumoniae* (MIC: 0.25-1 μg/mL). In particular, the most active compound **8f** is 8-128 fold more potent than the reference drugs MX, GM, CP and LV against methicillin-resistant *Staphylococcus aureus* 10-05 and *Streptococcus hemolyticus* 1002 and 2-64 fold more against methicillin-sensitive *Staphylococcus aureus* 10-03 and 10-04.

## 3. Experimental

### 3.1. General

All chemical reagents and solvents used in this study were purchased from Beihua Fine Chemicals Company (Beijing, China). Melting points were determined by X-5 digital display binocular microscope for melting-point tests (Gongyi, China) and are uncorrected. ^1^H-NMR spectra were recorded on a Varian Mercury-400 spectrometer using tetramethylsilane as internal standard. Electron spray ionization (ESI) mass spectra and high resolution mass spectra (HRMS) were recorded on a MDSSCIEX Q-Tap mass spectrometer. Merck silica gel ART5554 60F254 plates were used for analytical TLC. Column chromatography was carried out on silica gel HG/T2354-92 made in Haiyang Chemical Company (Qingdao, China).

*tert-Butyl 3-cyano-4-oxopiperidine-1-carboxylate* (**4**). A mixture of ethyl 3-aminopropanoate hydrochloride (**1**, 184.3 g, 1.2 mol), sodium hydroxide (48.0 g, 1.2 mol) and methanol (600 mL) was stirred for 0.5 h, and then acrylonitrile (79.5 g, 1.5 mol) was added dropwise over a period of 40 min at room temperature. The reaction mixture was heated to 65 °C and stirred for 3 h to give the secondary amine **2**, which was pure enough to be used for the next step without further purification. To the reaction mixture containing the amine **2 **was added (Boc)_2_O (218.0 g, 1.0 mol) at room temperature, and the mixture was stirred at 55–60 °C for 1 h and filtered. The filtrate was concentrated under reduced pressure. The residue was diluted with ethyl acetate (400 mL), washed with water and then saturated brine, dried over anhydrous sodium sulfate and filtered. The filtrate was concentrated under reduced pressure to afford the cyano ester **3 ** as a colorless oil. To a refluxing suspension of sodium hydride (70%, 41.1 g, 1.2 mol) in dry toluene (600 mL) a solution of the cyano ester **3** (270 g, 1.0 mol) dissolved in dry toluene (200 mL) was added dropwise over a period of 0.5 h. The reaction mixture was stirred for 1 h at the same temperature, cooled to room temperature and then water (400 mL) was added slowly. The aqueous layer was separated and adjusted to pH 7 with 10% acetic acid. The solid obtained was filtered, washed twice with water and dried in *vacuo* to give the title compound **4 **(224.2 g, 83%) as a yellow solid, m.p.: 97 °C 99 °C. ^1^H-NMR (CDCl_3_) δ 1.47 (9H, s, Boc-9H), 2.34–2.36 (2H, m, C_5_-H), 3.56–3.59 (2H, m, C_6_-H), 4.16–4.18 (2H, m, C_2_-H), 6.82 (1H, s, C_3_-H); MS (ESI) *m/z* 223 (M-H)^+^.

*tert-Butyl 3-amino-4,5,6,7-dihydro-2-methyl-2H-pyrazolo[4,3-c]pyridine)-carboxylate* (**5**). A solution of *tert*-butyl 3-cyano-4-oxopiperidine-1-carboxylate (**4**, 2.2 g, 10.0 mmol) and methylhydrazine (0.5 g, 11.0 mmol) dissolved in ethanol (50 mL) was stirred for 12 h at room temperature to give the Boc-protected bicyclic compound **5** (1.5 g, 60%) as a yellow solid, m.p.:163–165 °C. ^1^H-NMR (CDCl_3_) δ 1.47 (9H, s, Boc-9H), 2.67–2.70 (2H, m, C_7_-H), 3.65 (2H, br., C_6_-H), 3.71 (3H, s, N-CH_3_), 4.27 (2H, br., C_4_-H); MS (ESI) *m/z* 253 (M+H)^+^.

*4,5,6,7-Tetrahydro-2-methyl-2H-pyrazolo[4,3-c]pyridin-3-amine hydrochloride* (**6**). Dried hydrogen chloride gas was bubbled through a solution of compound **5** (1.5 g, 6.0 mmol) in methanol (20 mL) at room temperature for 0.5 h, and then stirred for another 0.5 h at the same temperature. The resulting solid was collected by suction, and dried in *vacuo* to give the title compound **6 **(0.6 g, 53%) as a yellow solid, mp: 252–254 °C. ^1^H-NMR (DMSO-*d_6_*) δ 2.72–2.75 (m, 2H, C_7_-H), 3.30–3.32 (m, 2H, C_6_-H), 3.53 (s, 3H, N-CH_3_), 3.86–3.88 (m, 2H, C_4_-H)；MS (ESI) *m/z* 153 (M+H)^+^.

*General procedure for the synthesis of 7-(3-amino-6,7-dihydro-2-methyl-2H-pyrazolo-[4,3-c]- pyridin-5(4H)-yl)fluoroquinolone derivatives* **8a-i**: A mixture of **7**(1.0 mmol), **6 **(1.5 mmol), triethylamine (8.0 mmol) and dry acetonitrile (20 mL) was stirred at 25~60 °C under an atmosphere of nitrogen for 1~48 h. The resulting solid was collected by suction, and dried in *vacuo* to give the title compounds **8a-i**.

*7-(3-Amino-6,7-dihydro-2-methyl-2H-pyrazolo[4,3-c]pyridin-5(4H)-yl)-1-cyclopropyl-6-fluoro-4-oxo-1,4-dihydro-1,8-naphthyridine-3-carboxylic acid* (**8a**). White solid (0.29 g, 73%), m.p.: >300 °C. ^1^H-NMR (DMSO-*d_6_*) δ 1.07–1.30 (m, 4H, 2CH_2_), 2.69–2.72 (m, 2H, C_7’_-H), 3.45 (s, 3H, N-CH_3_), 3.71–3.74 (m, 1H, CH), 4.02–4.05 (m, 2H, C_6’_-H), 4.68 (s, 2H, C_4’_-H), 8.06 (d, *J* = 13.6 Hz, 1H, C_5_-H), 8.82 (1H, s, C_2_-H); HRMS (ESI) m/z (M+H)^+^: calcd for C_19_H_20_FN_6_O_3_^+^: 399.15809; found: 399.15777.

*7-(3-Amino-6,7-dihydro-2-methyl-2H-pyrazolo[4,3-c]pyridin-5(4H)-yl)-1-(2,4-difluorophenyl)-6-fluoro-4-oxo-1,4-dihydro-1,8-naphthyridine-3-carboxylic acid* (**8b**). Yellow solid (0.37 g, 78%), m.p.: >300 °C. ^1^H-NMR (DMSO-*d_6_*) δ 2.07–2.34 (m, 2H, C_7’_-H), 3.41 (s, 3H, N-CH_3_), 4.491–4.495 (m, 2H, C_6’_-H), 5.10 (s, 2H, C_4’_-H), 7.34–7.39 (m, 1H, Ph-H), 7.60–7.66 (m, 1H, Ph-H), 7.79–7.85 (m, 1H, Ph-H), 8.14 (d, *J* = 13.2 Hz, 1H, C_5_-H), 8.85 (1H, s, C_2_-H); HRMS (ESI) m/z (M+H)^+^: calcd for C_22_H_18_F_3_N_6_O_3_^+^: 471.13925; found: 471.13972.

*7-(3-Amino-6,7-dihydro-2-methyl-2H-pyrazolo[4,3-c]pyridin-5(4H)-yl)-1-ethyl-6,8-difluoro-4-oxo-1,4-dihydroquinoline-3-carboxylic acid* (**8c**). White solid (0.21 g, 52%), m.p.: >300 °C. ^1^H-NMR (DMSO-*d_6_*) δ 1.45 (t, *J* = 6.8 Hz, 3H, CH_2_CH_3_), 2.63 (br., 2H, C_7’_-H), 3.47 (s, 3H, N-CH_3_), 4.21 (s, 2H, C_6’_-H), 4.59–4.60 (m, 2H, CH_2_CH_3_), 5.09 (s, 2H, C_4’_-H), 7.86 (d, *J* = 12.0 Hz, 1H, C_5_-H), 8.92 (s, 1H, C_2_-H); HRMS (ESI) m/z (M+H)^+^: calcd for C_19_H_20_F_2_N_5_O_3_^+^: 404.15342; found: 404.15339.

*7-(3-Amino-6,7-dihydro-2-methyl-2H-pyrazolo[4,3-c]pyridin-5(4H)-yl)-1-(2-fluoroethyl-6,8-difluoro-4-oxo-1,4-dihydroquinoline-3-carboxylic acid* (**8d**). Yellow solid (0.27 g, 64%), m.p.: >300 °C. ^1^H-NMR (DMSO-*d_6_*) δ 2.60–2.62 (m, 2H, C_7’_-H), 3.45 (s, 3H, N-CH_3_), 3.52 (br., 2H, C_6’_-H), 4.19 (s, 2H, C_7’_-H), 4.83–5.07 (m, 4H, CH_2_CH_2_F), 7.88 (d, *J* = 12.4 Hz, 1H, C_5_-H), 8.85 (1H, s, C_2_-H); HRMS (ESI) m/z (M+H)^+^: calcd for C_19_H_19_F_3_N_5_O_3_^+^: 422.14400; found: 422.14732.

*7-(3-Amino-6,7-dihydro-2-methyl-2H-pyrazolo[4,3-c]pyridin-5(4H)-yl)-1-cyclopropyl-6,8-difluoro-4-oxo-1,4-dihydroquinoline-3-carboxylic acid* (**8e**). The title compound was obtained as a yellow solid (0.23 g, 56%), m.p.: >300 °C. ^1^H-NMR (DMSO-*d_6_*) δ 1.18–1.33 (m, 4H, 2CH_2_), 2.88–2.91 (m, 2H, C_7’_-H), 3.62 (br., 2H, C_6’_-H), 3.68 (s, 3H, N-CH_3_), 4.00–4.01 (m, 1H, CH), 4.32 (s, 2H, C_4’_-H), 7.92 (d, *J* = 11.2 Hz, 1H, C_5_-H), 8.78 (s, 1H, C_2_-H); HRMS (ESI) m/z (M+H)^+^: calcd for C_20_H_20_F_2_N_5_O_3_^+^: 416.15342; found: 416.15416.

*7-(3-Amino-6,7-dihydro-2-methyl-2H-pyrazolo[4,3-c]pyridin-5(4H)-yl)-1-cyclopropyl-6-fluoro-8-difluoromethoxyl-4-oxo-1,4-dihydroquinoline-3-carboxylic acid* (**8f**). Yellow solid (0.31 g, 68%), m.p.: >300 °C. ^1^H-NMR (DMSO-*d_6_*) δ 1.02–1.19 (m, 4H, 2CH_2_), 2.61–2.64 (m, 2H, C_7’_-H), 3.46 (s, 3H, N-CH_3_), 3.50–3.51 (m, 1H, CH), 4.07–4.15 (m, 2H, C_6’_-H), 5.11 (s, 2H, C_4’_-H), 6.92 (t, *J* = 73.6 Hz, 1H, OCHF_2_), 7.91 (d, *J* = 12.4 Hz, 1H, C_5_-H), 8.76 (s, 1H, C_2_-H); HRMS (ESI) m/z (M+H)^+^: calcd for C_21_H_21_F_3_N_5_O_4_^+^: 464.15456; found: 464.15348.

*7-(3-Amino-6,7-dihydro-2-methyl-2H-pyrazolo[4,3-c]pyridin-5(4H)-yl)-1-cyclopropyl-6-fluoro-8-methoxyl-4-oxo-1,4-dihydroquinoline-3-carboxylic acid* (**8g**). Yellow solid (0.14 g, 32%), m.p.: >300 °C. ^1^H-NMR (DMSO-*d_6_*) δ 1.01–1.15 (m, 4H, 2CH_2_), 2.62–2.65 (m, 2H, C_7’_-H), 3.30 (s, 3H, N-CH_3_), 3.42–3.64 (m, 2H, C_6’_-H), 3.70 (s, 3H, OCH_3_), 4.16 (s, 2H, C_4’_-H), 4.17–4.18 (m, 1H, CH), 7.75 (d, *J* = 12.0 Hz, 1H, C_5_-H), 8.69 (s, 1H, C_2_-H); HRMS (ESI) m/z (M+H)^+^: calcd for C_21_H_23_FN_5_O_4_^+^: 428.17341; found: 428.17379.

*7-(3-Amino-6,7-dihydro-2-methyl-2H-pyrazolo[4,3-c]pyridin-5(4H)-yl)-1-cyclopropyl-5-amino-6-fluoro-8-methoxyl-4-oxo-1,4-dihydroquinoline-3-carboxylic acid* (**8h**). Yellow solid (0.08 g, 17%), m.p.: >300 °C. ^1^H-NMR (DMSO-*d_6_*) δ 0.93–1.11 (m, 4H, 2CH_2_), 2.09 (br., 2H, C_7’_-H), 2.64 (br., 2H, C_6’_-H), 3.46 (s, 3H, N-CH_3_), 3.52 (br., 1H, CH), 3.62 (s, 3H, OCH_3_), 4.17 (s, 2H, C_4’_-H), 8.67 (s, 1H, C_2_-H); HRMS (ESI) m/z (M+H)^+^: calcd for C_21_H_23_FN_6_O_4_^+^: 443.18431; found: 443.18522.

*9-fluoro-3(S)-methyl-10-(3-amino-6,7-dihydro-2-methyl-2H-pyrazolo[4,3-c]pyridine-5(4H)-yl)-7-oxo-2,3-dihydro-7H-pyrrido[1,2,3-d,e][1,4]benzoxazine-6-carboxylic acid* (**8i**). Yellow solid (0.17 g, 41%), m.p.: >300 °C. ^1^H-NMR (DMSO-*d_6_*) δ 1.45 (d, *J* = 6.8 Hz, 3H, CH_3_), 2.55–2.65 (m, 2H, C_7’_-H), 3.30 (s, 3H, N-CH_3_), 4.13–4.16 (m, 2H, C_6’_-H), 4.34–4.37 (m, 1H, OCH_2_CHN), 4.46–4.49 (m, 2H, OCH_2_CHN), 5.01 (s, 2H, C_4’_-H), 7.60 (d, *J* = 12.4 Hz, 1H, C_5_-H), 8.95 (s, 1H, C_2_-H); HRMS (ESI) m/z (M+H)^+^: calcd for C_20_H_21_FN_5_O_4_^+^: 414.15776; found: 414.15497.

### 3.2. Antibacterial activity

Compounds **8a-i** were evaluated for their *in vitro* antibacterial activity in comparison to several reference drugs using the conventional agar-dilution method. Drugs (10.0 mg) were dissolved in 0.1 N sodium hydroxide (10 mL). Further progressive twofold serial dilution with melted Mueller-Hinton agar was performed to obtain the required concentrations of 128, 64, 32, 16, 8, 4, 2, 1, 0.5, 0.25, 0.125, 0.06, and 0.03 μg/mL. Petri dishes were incubated with 104 colony forming units (cfu) and incubated at 35 °C for 18 h. The MIC was the lowest concentration of the test compound, which resulted in no visible growth on the plate.

## 4. Conclusions

We report herein the synthesis of a series of novel 7-(3-amino-6,7-dihydro-2-methyl-2*H*- pyrazolo[4,3-c]pyridin-5(4*H*)-yl)-6-fluoro-4-oxo-1,4-dihydroquinoline-3-carboxylic acid derivatives. The antibacterial activities of the newly synthesized compounds were evaluated. The test results reveal that most of the target compounds have good growth inhibiting activity against methicillin-resistant *Staphylococcus epidermidis* (MRSE) (MIC: 0.25–4 μg/mL) and *Streptococcus pneumoniae* (MIC: 0.25–1 μg/mL). In addition, compound **8f** is 8–128 fold more potent than the reference drugs MX, GM, CP and LV against methicillin-resistant *Staphylococcus aureus* 10-05 and *Streptococcus hemolyticus* 1002 and 2–64 fold more active against methicillin-sensitive *Staphylococcus aureus* 10-03 and 10-04. However, all of them display generally rather weak potency against the tested Gram-negative strains. The reduced activity might be due to the conjugation effect between the introduced new double bond and the amino group possessing a lone pair of electrons on the nitrogen, hindering the amino group from participating in hydrogen bonding with the drug target, as is known to be the case of quinolone with DNA gyrase. 
